# Use veno-venous extra corporeal membrane oxygenation in elderly patients with post-cardiotomy hypoxia: the changing paradigm of respiratory support in adult respiratory distress syndrome

**DOI:** 10.1186/s13019-019-0833-y

**Published:** 2019-01-14

**Authors:** Sara Volpi, Federico Sertic, Kamen Valchanov, Ravi De Silva

**Affiliations:** Department of Cardiothoracic Surgery, Royal Papworth Hospital, Cambridge, CB23 3RE UK

**Keywords:** Veno-venous extracorporeal membrane oxygenation (VV-ECMO), Acute respiratory distress syndrome (ARDS), cardiac surgery

## Abstract

**Background:**

Veno-venous extracorporeal membrane oxygenation (VV-ECMO) support for ARDS treatment after cardiac surgery has progressed remarkably in the last 20 years. However, one of the limitations of a successful recover is age, being a powerful predictor of mortality.

**Case presentation:**

In this case report we discuss a 78-year-old man who underwent aortic valve and aortic root replacement. The postoperative period was complicated by ARDS following aspiration pneumonia treated with VV-ECMO weaned after 6 days. At two-year follow up, the patient made an excellent recover, being the second oldest person to survive VV-ECMO following cardiac surgery in the world.

**Conclusion:**

In the literature there is no consensus regarding a specific age limit and results, in the use of ECMO in the elderly are scarce and inconsistent. We do not think advanced age is a contraindication to the use of ECMO.

## Background

Acute respiratory distress syndrome (ARDS) is a life-threatening complication that occasionally occurs after cardiac surgery. It can lead to rapidly progressive respiratory failure and has a high mortality rate [1]. Over the last 20 years, second generation veno-venous extracorporeal membrane oxygenation (VV-ECMO) support has emerged and progressed remarkably, becoming a valuable alternative therapy to provide good gas exchange and facilitate the recovery process in ARDS [2]. One of the limitations of a successful recover is age, being a powerful predictor of mortality [3]. We present the second oldest person to survive VV-ECMO following cardiac surgery in the world.

## Case presentation

**Fig. 1:**
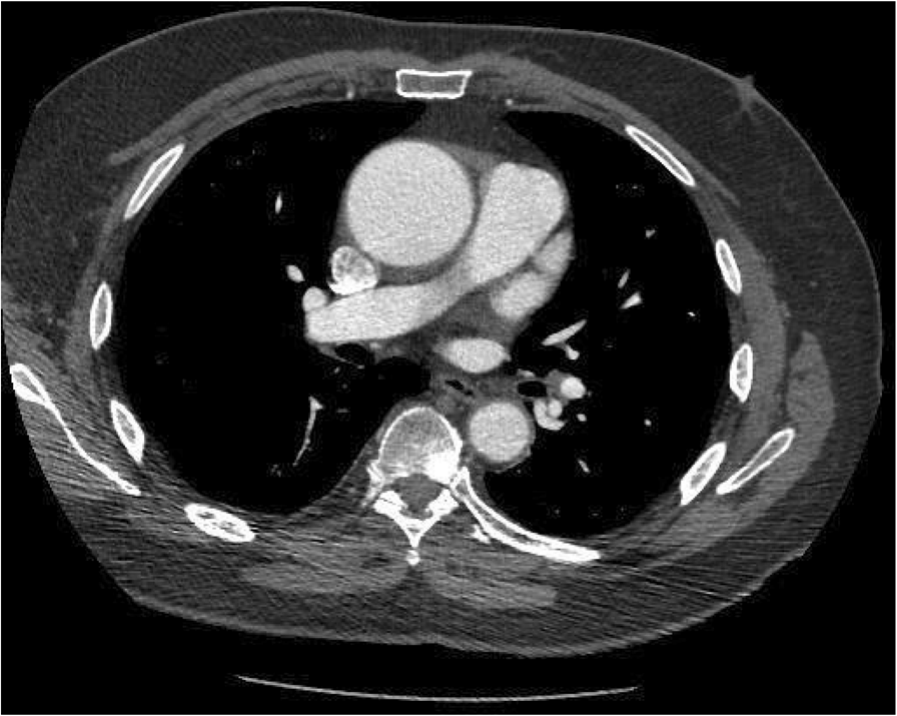
CT Aorta showing aortic dilatation. No signs of dissection

**Fig. 2:**
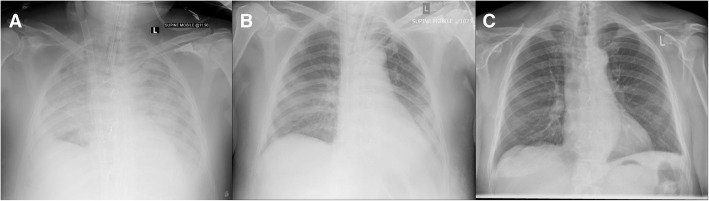
Chest X-Rays performed: (**a**) after VV-ECMO insertion: patchy airspace of opacification bilaterally. Features in keeping with ARDS; (**b**) 6 days after VV-ECMO insertion, immediately before weaning: significant improvement in the bilateral perihilar pulmonary infiltrates; (**c**) at three-months follow up: heart size normal, lungs and pleural spaces clear

A 78-year-old man with a history of hypertension, atrial fibrillation and previous left lung tuberculosis presented with shortness of breath (NYHA II) and chest pain (CCS II-III). An echocardiogram showed calcified bicuspid aortic valve with mild regurgitation and a dilated ascending aorta with preserved biventricular function. Cardiac CT scan confirmed maximal ascending aorta dilatation of 5.2 cm (Fig. 1). The coronary angiogram showed no coronary artery disease. The Logistic EuroSCORE was 14.74%. Therefore, he underwent aortic valve and aortic root replacement with left atrial appendage excision using a 25 mm Hancock II bioprosthesis and a 28 mm Hemashield graft. The patient was weaned from cardiopulmonary bypass ventricularly paced with Noradrenaline. The bypass time was 116 min with a cross clam time of 100 min using a retrograde cardioplegia technique. Protamine was used to reverse the heparin effect. The intraoperative transoesophageal echocardiogram was satisfactory and the patient was transferred to the Intensive Care Unit (ICU) in stable condition. The day after surgery he was brought back to theatre for bleeding. Despite several bronchoscopies with removal of large amounts of clots, the patient remained hypoxic (Table 1). ARDS following aspiration pneumonia with pulmonary hemorrhage was diagnosed based on the Berlin definition. Therefore, he was supported with VV-ECMO, according to NICE and Extracorporeal Life Support Organization (ELSO) Guidelines [4], on day 4 after surgery together with continuous veno-venous hemofiltration (CVVH) for refractory metabolic acidosis. Single VV-ECMO cannulation with bi-caval dual-lumen cannula (Avalon ELITE™, Avalon Laboratories, USA) was performed percutaneously through the right internal jugular vein and a 4-l flow was established (Fig. 2a). In the following days, he became hemodynamically more stable and the gas exchange and CXR improved consistently (Fig. 2b). VV-ECMO and CVVH were weaned off in 6 days. A CT head, thorax, abdomen and pelvis done as per ECMO protocol ruled out intracranial pathologies, features of colitis and gut ischaemia and chest pathologies. The total patient stay in ICU was 22 days. He made a good recovery and he was discharged to a secondary facility for further rehabilitation. At two-year follow up, the patient made excellent progress, being one if the oldest person to survive VV-ECMO following cardiac surgery in the world. His ECG shows sinus rhythm and the chest X-ray is unremarkable (Fig. 2c).

**Table 1:** Ventilation setting and arterial blood gas showing metabolic acidosis

Variable	Value
Ventilation Mode	Pressure Regulated Volume Control (PRVC)
Respiratory Rate	14 bpm
PEEP	12 cm H_2_O
PIP	30 cm H_2_O
Tidal Volume	683 mL
FiO2	100%
PaO2	6.41 kPa ↓
SaO2	85.1% ↓
PaCO2	6.23 kPa
pH	7.34 ↓
HCO3	24.5 Mmol/l ↑
Lactate	2.6 mEq/l ↑

## Discussion and conclusion

Current indications and contraindications for appropriate use of Extracorporeal-Membrane-Oxygenation (ECMO) are set by the Extracorporeal-Life-Support-Organization (ELSO) [4]. In the ELSO guidelines advanced age is reported as a relative contraindication even though a specific threshold is not described [3]. The largest available trial (CESAR trial) on the use of VV-ECMO for the treatment of ARDS did not include any patients over the age of 65 years or any post-operative patients [5]. The postcariotomy veno-venous ECMO area is not well explored in the literature. In particular, the only available literature that reports the use of VV-ECMO following cardiac surgery for the treatment of ARDS is based on 2 case series [1, 2]. They both concluded that the VV-ECMO is a feasible option in the treatment of ARDS following cardiac surgery when conventional conservative management fails. Nakamura et al. presented a series of 11 patients with a survival to hospital discharge of 63.6% and the non-survivors were significantly older (*p* < 0.01) [1]. Song et al. presented a series of 13 patients with a survival to hospital discharge of 53.8% with the oldest survival being 74-years-old [2].

These patients differ form veno-venous ECMO for respiratory failure due to other causes in few ways. First of all, the cause of respiratory failure is known (such as aspiration pneumonia in our case). Moreover, the patients are already in a centre for advanced respiratory failure management, and institution of ECMO can be done early. Furthermore, the cardiac function is normal, unlike elderly patients from the community who may have cardiac disease. Finally, co-morbidities like malignancy have already been ruled out.

We present the case of a 78-years-old man who underwent AVR + ARR and developed severe ARDS post-operatively requiring VV-ECMO. He survived to hospital discharge and at 2-years follow-up he is doing remarkably well, being the second oldest survivor, to the best of our knowledge, to VV-ECMO after cardiac surgery in the world.

In conclusion, advanced age has been described as a relative contraindication to the use of ECMO. However, in the literature there is no consensus regarding a specific age limit and results in the use of ECMO in the elderly are scarce and inconsistent. We described a case of elderly patient who benefitted from extracorporeal support. Although mortality is higher in the elderly, for carefully selected postcardiotomy patients, ECMO support can be valuable and lead to excellent recovery.

## Abbreviations

VV-ECMO: Veno-venous extracorporeal membrane oxygenation
ARDS: Acute respiratory distress syndrome
ICU: Intensive Care Unit
CVVH: continuous veno-venous hemofiltration
ELSO: Extracorporeal-Life-Support-Organization
AVR: aortic valve replacement
ARR: aortic root replacement
